# Photobacteriosis: Prevention and Diagnosis

**DOI:** 10.1155/2014/793817

**Published:** 2014-05-28

**Authors:** Francesca Andreoni, Mauro Magnani

**Affiliations:** Department of Biomolecular Science, Biotechnology Section, University of Urbino “Carlo Bo”, Via Arco d'Augusto 2, 61032 Fano, Italy

## Abstract

Photobacteriosis or fish pasteurellosis is a bacterial disease affecting wild and farm fish. Its etiological agent, the gram negative bacterium *Photobacterium damselae* subsp. *piscicida*, is responsible for important economic losses in cultured fish worldwide, in particular in Mediterranean countries and Japan. Efforts have been focused on gaining a better understanding of the biology of the pathogenic microorganism and its natural hosts with the aim of developing effective vaccination strategies and diagnostic tools to control the disease. Conventional vaccinology has thus far yielded unsatisfactory results, and recombinant technology has been applied to identify new antigen candidates for the development of subunit vaccines. Furthermore, molecular methods represent an improvement over classical microbiological techniques for the identification of *P. damselae* subsp. *piscicida* and the diagnosis of the disease. The complete sequencing, annotation, and analysis of the pathogen genome will provide insights into the pathogen laying the groundwork for the development of vaccines and diagnostic methods.

## 1. Introduction

Photobacteriosis or fish pasteurellosis is a septicemia caused by the gram negative, halophilic bacterium* Photobacterium damselae* subsp.* piscicida*, a member of the Vibrionaceae family, that shares its species epithet with* Photobacterium damselae* subsp.* damselae* [1]. Photobacteriosis is considered one of the most dangerous bacterial diseases in aquaculture worldwide due to its wide host range, high mortality rate, and ubiquitous distribution [2]. The pathogen is able to infect a wide variety of marine fish, including the yellowtail (*Seriola quinqueradiata*) in Japan, gilthead sea bream (*Sparus aurata*), sea bass (*Dicentrarchus labrax*), and sole (*Solea senegalensis* and* Solea solea*) in Europe, striped bass (*Morone saxatilis*), white perch (*Morone americana*), and hybrid striped bass (*Morone saxatilis *(*Morone chrysops*)) in the USA, cobia (*Rachycentron canadum*) in Taiwan, and golden pompano (*Trachinotus ovatus*) in China [3–5].

Differences in susceptibility to the disease have been described on the basis of fish age. Larvae and juveniles are more susceptible to photobacteriosis, and acute infection induces 90–100% mortality of juvenile sea bream, whereas fish over 50 g are more resistant due to more efficient phagocytosis and killing of the bacteria by neutrophils and macrophages [6]. Bacteria that reside in different tissues and inside phagocytes cause chronic and acute forms of photobacteriosis. In its acute form, multifocal necrosis is present in the liver, spleen, and kidney and bacteria accumulate freely in phagocytes, capillaries, and interstitial spaces. Chronic lesions in the internal organs are characterized by the presence of white tubercles about 0.3–0.5 mm in diameter [7].

Adherence and invasive capacities are essential in the first stage of infection [3].* P. damselae* subsp.* piscicida* has been reported to be weakly or moderately adherent and invasive in various fish cell lines but has shown a high binding capacity to fish intestines [8]. The adherence seems to be mediated by a protein or glycoprotein receptor of the bacterial cell surface, and the internalization of the bacteria occurs through an actin microfilament-dependent mechanism [8], with cell metabolism playing an active role [9]. However, the precise nature of the mechanism responsible for adherence and interaction with host cell receptors and virulence factors contributing to the invasion of fish nonphagocytic cells is still unknown [9].

Several virulence mechanisms of* P. damselae* subsp.* piscicida* have been described. The polysaccharide capsular material plays an important role in the pathogenesis of the bacterium, conferring resistance to serum killing and increasing fish mortality [10]. Furthermore, the intracellular survival of the pathogen is likely to confer protection against specific and nonspecific host defenses and exogenous antimicrobial agents including antibiotics [8]. Extracellular products with phospholipase, cytotoxic, and hemolytic activities may account for the damage to the infected cells, the consequent release of the microorganisms, and the invasion of adjacent cells. In particular, a key pathogenicity factor of* P. damselae* subsp.* piscicida* is an exotoxin, the plasmid-encoded apoptosis-inducing protein of 56 kDa (AIP56), abundantly secreted by virulent strains and responsible for apoptogenic activity against sea bass macrophages and neutrophils in acute fish photobacteriosis [11]. The AIP56 toxin is a zinc-metalloprotease that acts by cleaving NF-*κ*B p-65, with the catalytic activity located in the N-terminal domain and the C-terminal domain involved in binding and internalization into the cytosol of target cells [12]. The AIP56 induces activation of caspases 8, 9, and 3, loss of mitochondrial membrane potential, release of cytochrome c into the cytosol, and overproduction of ROS, suggesting activation of both extrinsic and intrinsic apoptotic pathways [13]. Through the activation of the cell death process involving macrophages and neutrophils, the pathogen is able to subvert the immune defenses of the host and to produce infectious disease.

Another important virulence mechanism of* P. damselae* subsp.* piscicida* is the acquisition of iron from its host by using high-affinity iron-binding siderophores, low molecular weight iron-chelating molecules that interact with bacterial membrane receptors to transport iron into the bacterium [14]. Furthermore,* P. damselae* subsp.* piscicida* is able to acquire iron from hemin and hemoglobin as unique iron sources* in vitro* [14], and iron limitation results in an increased binding of hemin in virulent strains [15]. The heme uptake of the bacterium includes a TonB system to transport heme into the cytoplasm and an ATP-binding cassette (ABC) system to drive it across the cytoplasmic membrane [16, 17].

Little is known about the fish immune response to the bacterium and the factors responsible for its failure to protect against* P. damselae* subsp.* piscicida.* A transcriptomic approach has recently been applied to elucidate the early immune responses of juvenile gilthead sea bream to* P. damselae* subsp.* piscicida* infection. A rapid recognition of the pathogen is shown by the upregulation of lectins, peptides with antimicrobial activity, chemokines, and chemokine receptors, as well as protein of iron and the heme metabolism as a response against bacteria that are dependent on iron. However, this defensive reaction can be either beneficial or devastating to the host [18]. Moreover, the upregulation of genes with highly specialized suppressive functions has been observed indicating an active suppression of immunity that can be induced by the host to reduce tissue damages or by the pathogen to evade the host response [18].

## 2. Prevention of* P. damselae* subsp.* piscicida* Infection

Antibiotics have been the first line of defense in fish aquaculture to control photobacteriosis outbreaks, but after only a few years the pathogen acquired resistance to various antibiotics. In fact, different transferable genetic elements (R plasmids) carrying genes for resistance against kanamycin, sulphonamide, tetracycline [19–22], ampicillin [22, 23], chloramphenicol [22, 24], florfenicol [25], and erythromycin [26] have been documented in* P. damselae* subsp.* piscicida*. Differences in the geographic distribution of multidrug transferable elements have been observed among several strains collected in Japan and United States [22, 27]. Furthermore, the intracellular parasitism of* P. damselae* subsp.* piscicida* within macrophages undermines the effectiveness of chemotherapy.

Taking into account all of these issues, research has been focused on the development of effective vaccines to prevent photobacteriosis and reduce the use of antibiotics in fish farming with benefits at biological and environmental point [28]. Conventional* P. damselae* subsp.* piscicida* vaccines are based on inactivated products containing cellular (heat-o formalin killed bacteria) and soluble antigens (LPS and ribosomal formulations) for immersion and injection administration (Table 1). However, they appear to be ineffective in protecting against pasteurellosis [2, 3, 28–38]. Bacterins overexpressing a 97 kDa OMP and a 52 kDa ECP protein, involved in the internalization of the bacterium, are reported to be effective in both sea bass and yellowtail when delivered by immersion eliciting a strong antibody response in the gills and mucosae that may block pathogen entry and colonization [2]. However, the only commercially available vaccine is an ECP-enriched bacterin preparation that has been employed in several European countries with mixed results ranging from good in Spain, Turkey, and Greece to poor in Italy [28, 39]. The recommended vaccination protocol consists of two bath immersions at monthly intervals starting at the larval stage when the fish is 50 mg and an oral booster immunization when fish reaches 2 g body weight [3].

Recombinant DNA technology and biotechnological approaches have thus far been used to a very limited extent for the development of bacterial vaccines for fish and effective preventive measures against fish pasteurellosis do not yet exist. Studies on the development of subunit vaccines have recently been reported in cobia from a Taiwan* P. damselae* subsp.* piscicida* isolate [4]. Immunoproteomics, using western blotting on protein analyzed with 2DE and LC-MS/MS to isolate immune-reactive proteins, has been applied to identify* P. damselae* subsp.* piscicida* antigens that were then cloned and produced as recombinant proteins. In particular, three antigens were shown to induce a protective effect in cobia and therefore were reported as potential vaccine candidates for the development of a subunit vaccine against the pathogen. However, the protection of these vaccine candidates has not been investigated in other fish species, where* P. damselae* subsp.* piscicida* causes serious disease and high mortality, and against other* P. damselae* subsp.* piscicida* isolates [4]. Moreover, antigen combinations were studied revealing that bivalent subunit vaccines may achieve a better efficiency than monovalent or trivalent antigens [41].

In our laboratory a biotechnological approach based on the* reverse vaccinology* has been applied to design a vaccine against fish pasteurellosis [40]. New genomic sequences of* P. damselae* subsp.* piscicida* were the starting point for bioinformatic analysis aiming to identify new proteins localized on the bacterial surface. In fact, the primary condition in selecting a bacterial protein as a vaccine candidate is its cellular localization. Cytosolic proteins are unlikely to be immunological targets, whereas surface exposed and secreted proteins are more easily accessible to the host immune system [47].* In vitro* screening of the* in silico* selected vaccine candidates by an inhibition adherence assay revealed that immunoglobulins from mice immunized with one of the recombinant vaccine candidates were able to affect the adherence of* P. damselae* subsp.* piscicida* to fish epithelial cells. The candidate antigen, found to be involved in the adherence and internalization of* P. damselae* subsp.* piscicida* in CHSE-214 cells, was predicted* in silico* as likely lipoprotein with outer membrane localization. The N-terminal signal peptide of 20 amino acids contains the lipobox motif, 2 positively charged residues within the first 7 amino acids and a transmembrane helix of 10 residues. A database search revealed homology with hypothetical proteins and no putative conserved domain; therefore, no putative biological role could be assigned to this lipoprotein. Vaccination and challenge experiments in a laboratory trial indicated that immunization of sea bass with the recombinant antigen induced the production of specific antibodies and conferred protection against* P. damselae* subsp.* piscicida* challenge [40].* In vivo* long persistence of lipoprotein antibodies was obtained with a single antigen administration in agreement with Ho et al. [4] who reported that multiple administrations do not increase protection in fish. The recombinant lipoprotein is potentially able to protect sea bass against* P*.* damselae* subsp.* piscicida* and could be an interesting candidate for the design of a recombinant vaccine against photobacteriosis. However, protection efficacy over time, increasing doses of the antigen, and its use in combination with different adjuvants must be further investigated in field experiments.

Due to the inconsistency of effective measures to prevent photobacteriosis, research has also focused on alternative methods to control the disease. Such methods include probiotics, to be applied in aquaculture to improve health, and a strain of* Pediococcus pentosaceus*, a lactic acid bacterium isolated from the intestine of adult cobia, has been investigated for its probiotic potential [48]. The acidic pH derived from metabolic acids in lactic acid bacteria culture supernatant has been shown to inhibit* P. damselae* subsp.* piscicida* growth* in vitro*. Dietary supplementation with* P. pentosaceus* in cobia enhances the growth rate and respiratory burst of peripheral blood leukocytes in fed fish. Furthermore, lactic acid bacteria feeding increased the survival rate of cobia after* P. damselae* subsp.* piscicida* immersion challenge. The mechanism affording this protection is still unclear. Although feeding with lactic acid bacteria did not increase specific antibody response after the immunization of cobia with inactivated* P. damselae* subsp.* piscicida* vaccine, it heightened the synergic protection against* P. damselae* subsp.* piscicida* challenge by 22% and could be administered by itself as a probiotic or with vaccination [48].

Furthermore, selective breeding for fish strains genetically resistant to photobacteriosis constitutes a potential strategy to reduce the probability of disease outbreak and avoid the dramatic consequences of high mortality in fish farms [49]. Quantitative trait loci mapping is applied to detect the regions of the host genome that are associated with resistance to the disease and marker-assisted selection is a useful approach used in several aquaculture species [50–52].

A study investigating quantitative trait loci for resistance to fish pasteurellosis in the gilthead sea bream identified two significant quantitative trait loci, one affecting late survival and another impacting overall survival, and a potential marker for disease resistance [49]. The identification of phase-specific quantitative trait loci in gilthead sea bream supports the hypothesis of a biphasic defense response with a primary infection by experimental exposure to the pathogen and a secondary infection with bacteria released from moribund and dead fish [49, 53]. Results of quantitative trait loci, mapped by identifying regions of the genome that explain complex traits such as survival, could also be used to gain a better understanding of the mechanisms of disease resistance and defense response. Further insights might also be gained through comparative mapping with other species susceptible to photobacteriosis.

## 3. Identification of* P. damselae* subsp.* piscicida* and Diagnosis of Infection

Rapid diagnosis of fish photobacteriosis outbreaks is essential for proper management and effective control in aquaculture. Disease diagnosis is usually made using standard microbiological methods, based on pathogen culturing and isolation steps. Biochemical and serological confirmation is also necessary to characterize the bacterium and to discriminate between the two closely related subspecies,* piscicida* and* damselae* of* P. damselae*. The miniaturized system AIP20E is usually used for a presumptive identification of the* P. damselae* subsp.* piscicida*. Although* P. damselae* generally displays a unique code of 2005004 for the* piscicida* [54] and 2015004 for the* damselae* subspecies [1], some strains exhibit aberrant reactions that can lead to misleading results [55]. Hence, differentiation of the subspecies* P. damselae* subsp.* damselae* can be achieved when three or more positive results are obtained in the lysine decarboxylase (LDC) production, motility, nitrate reduction to nitrite, gas production from glucose, thiosulfate citrate bile salts-sucrose (TCBS-1) growth, and urease tests, because all these tests yield negative results for all* P. damselae* subsp.* piscicida* strains [55, 56]. Serological methods such as agglutination or the ELISA have also been developed and commercialized [3].

To overcome the problem of time-consuming and laborious procedures, in the last few years molecular methods have been developed in order to achieve an accurate and specific identification of* P. damselae* subsp.* piscicida* and a rapid diagnosis of photobacteriosis (Table 2). The point at issue is the strong similarity at the DNA level between the two subspecies that makes it difficult to identify sequences useful for designing a subspecies-specific method [3, 42, 57]. rRNA sequences have been considered for this purpose [42], but strong similarities have been detected both in the 16S, 23S, and 5S (>99%) and the intergenic spacer regions (98–99.5%) between the two subspecies of* P. damselae*. Moreover, the mosaic-like structure of the latter makes them unsuitable for diagnostic purposes [42, 58]. Only a PCR-based method at species level has been developed using the 16S sequences [42].

Integrated sets of methods combine the amplification of the capsular polysaccharide gene to identify the species* P. damselae* with an additional culture step on TCBS-1 agar to differentiate* P. damselae* subsp.* piscicida* from* P. damselae* subsp.* damselae* [44] or the amplification of two* P. damselae*-specific targets with restriction analysis of PCR products to obtain a unique digestion profile for* P. damselae* subsp.* piscicida* strains [46].

A multiplex PCR method based on the 16S rRNA and* ureC* genes has been proposed to discriminate between the two subspecies. The* ureC* gene is present in* P. damselae* subsp.* damselae* genome but is not found in* P. damselae* subsp.* piscicida* [1]. On the contrary, a* P. damselae* subsp.* piscicida*-specific target sequence, conserved among strains of different geographical origin but not shared by* P. damselae* subsp.* damselae*, has not yet been reported [42, 44].

An additional multiplex PCR protocol has been developed in our laboratory as a valid alternative to standard culture methods for the rapid and specific diagnosis of photobacteriosis in fish [43]. The gene coding for a penicillin binding protein 1A (GenBank accession number EU164926) was selected from a large-scale genome project as the PCR target for the identification of* P. damselae* subsp.* piscicida* because of several mismatches with the corresponding* P. damselae* subsp.* damselae* gene mainly clustered in the 3′ end of the gene. However, specificity analysis also indicated amplification of the target gene in two* P. damselae* subsp.* damselae* strains. This is due to the fact that a stronger sequence similarity to* P. damselae* subsp.* piscicida* than to other* P. damselae* subsp.* damselae* strains was found in these two* P. damselae* subsp.* damselae* strains. Hence, an additional PCR target, the* ureC* gene, lacking in the* P. damselae* subsp.* piscicida* genome, was introduced in the assay with the aim of differentiating each strain at the subspecies level together with an internal amplification control to obtain a clear distinction between truly negative and false negative results. The optimized multiplex PCR is able to correctly identify and discriminate both subspecies of* P. damselae* with a detection limit of 500 fg DNA, corresponding to 100 genomic units, twofold higher than that of immunodiagnostic systems (i.e., Bionor Aquaeia-Pp kit) [45].

## 4. Conclusions

Partial genome sequencing of several* P. damselae* subsp.* piscicida* strains has been previously reported [40, 59] and recently a draft of the complete genome sequence of* P. damselae* subsp.* piscicida* DI21 strain has been deposited in the public databases (GeneBank accession number PRJNA168653), but the complete gene annotation is not yet available. This information together with the comparative analysis of the genome sequence of different strains of* P. damselae* subsp.* piscicida* and* P. damselae* subsp.* damselae* will provide further insights laying the groundwork for the development of effective vaccines and diagnostic tools for the causative agent of fish pasteurellosis.

## Figures and Tables

**Table 1:** Overview of vaccines against *Photobacterium damselae* subsp. *piscicida. *

	Type of vaccine	Type of product	Vaccination procedure	Species	References
Lipoprotein	Recombinant subunit vaccine	Experimental	Injection	Sea bass	Andreoni et al. [40]

rHSP60, rENOLASE, and rGAPDH antigens, singles or in combination	Recombinant subunit vaccine	Experimental	Injection	Cobia	Ho et al. [4] and Ho et al. [41]

Formalin-killed bacterin overexpressing a 97 kDa OMP and 52 kDa ECP	Inactivated	Licensed	Immersion	Sea bass and yellowtail	Barnes et al. [2]

Formalin-killed bacterin with Escherichia coli LPS	Inactivated	Experimental	Immersion	Sea bream	Hanif et al. [38]

Live attenuated aroA mutant	Live attenuated	Experimental	Injection or immersion	Hybrid striped bass	Thune et al. [37]

Formalin-killed bacterin, ECP, and crude capsular polysaccharide (cCPS)	Inactivated	Experimental	Injection, immersion, and oral	Sea bass	Bakopoulos et al. [36]

LPS mixed with chloroform-killed bacterin	Inactivated	Experimental	Injection	Yellowtail	Kawakami et al. [35]

ECP-enriched formalin-inactivated bacterin	Inactivated	Commercialized	Immersion	Sea bass, sea bream, and sole	Magariños et al. [39]

Live attenuated bacteria	Live attenuated	Experimental	Immersion	Yellowtail	Kusuda and Hamaguchi [34]

Ribosomal antigens	Subunit vaccine	Experimental	Injection	Yellowtail	Kusuda et al. [33]

LPS formulation	Subunit vaccine	Experimental	Immersion and spray methods	Yellowtail	Fukuda and Kusuda [32]

Heat- and formalin-killed bacterin	Inactivated	Experimental	Immersion and oral	Yellowtail	Fukuda and Kusuda [29], Hamaguchi and Kusuda [30], and Kusuda and Salati [31]

**Table 2:** Methods for direct identification of *Photobacterium damselae* subsp. *piscicida. *

Assay	Target	Additional culture step	Specificity	Availability on the market	References
PCR-based detection method	*16S* gene	—	*P. damselae *		Osorio et al. [42]

Multiplex PCR assay	*16S* gene *ureC* gene	—	*P. damselae *subspecies		Osorio et al. [1]

Multiplex PCR assay	*Pbp-1A* gene *ureC* gene internal amplification control	—	*P. damselae *subspecies	*Photobacterium damselae*-PCR detection Kit by Diatheva	Amagliani et al. [43]

PCR technique and plating method	*cps* gene	TCBS-1 agar	*P. damselae *subspecies		Rajan et al. [44]

Enzyme immunoassay	Polyclonal antibodies against *P. damselae *subsp*. piscicida *	—	*P. damselae *subsp*. piscicida* ^a^	Aquaeia-Pp kit by BIONOR	Romalde et al. [45]

PCR-RFLP method	GenBank AY191120, AY191121 sequences	—	*P. damselae*, restriction analysis for subspecies identification		Zappulli et al. [46]

^a^Cross reactions with *P. damselae* subsp. *damselae* and *P. histaminum*.
